# Gender Differences in Suicide Prevention Responses: Implications for Adolescents Based on an Illustrative Review of the Literature

**DOI:** 10.3390/ijerph120302359

**Published:** 2015-02-23

**Authors:** Emma Hamilton, Bonnie Klimes-Dougan

**Affiliations:** 1 Educational Psychology Department, University of Texas at Austin, 1 University Station Austin, TX 78712, USA; 2 Department of Psychology, University of Minnesota, 75 East River Road Minneapolis, MN 55455, USA; E-Mail: klimes@umn.edu

**Keywords:** gender, suicide, prevention, programming, adolescents

## Abstract

Background: There are well-documented gender differences in adolescent suicidal behavior; death by suicide is more common in males, while nonfatal suicide attempts are more common among females. Over the past three decades, researchers have documented the effectiveness of a myriad of suicide prevention initiatives. However, there has been insufficient attention to which types of suicide prevention interventions are effective in changing attitudes and behaviors for young males and females. In this review of the literature, we consider common examples of primarily universal suicide prevention programs from three implementation settings: school-based, community-based, and healthcare-based. Our purpose is to delineate how the potential gender bias in such strategies may translate into youth suicide prevention efforts. Methods: Research in which gender was found to moderate program success was retrieved through online databases. Results: The results that feature programming effects for both males and females are provocative, suggesting that when gender differences are evident, in almost all cases, females seem to be more likely than males to benefit from existing prevention programming. Conclusions: We conclude by considering recommendations that may benefit males more directly. Implications for adolescent suicide prevention in particular are discussed. Personalization of suicide intervention is presented as a promising solution to reduce suicide rates.

## 1. Introduction

Suicide is a leading cause of death and a significant mental health problem worldwide [1,2]. Suicide is defined as death caused by self-directed injurious behavior with any intent to die as a result of the behavior [3]. Adolescence is a period of marked risk for suicidality [1]. For youth between the ages of 10 and 24, suicide is the third leading cause of death, significantly superseding the rate for adults aged 35 to 54 [4]. Males are more likely to die as a result of suicide: the male-to-female ratio of death by suicide is four to one in the U.S. [1,2,4].

Suicide attempt is defined as a non-fatal self-directed potentially injurious behavior with any intent to die as a result of the behavior; a suicide attempt may or may not result in injury [3]. In almost all regions of the world, nonlethal suicide attempts are more common in females [2]. Males are more likely to use more lethal means than females, partially accounting for the different pattern for suicide deaths and attempts [1,5]. Gender differences also exist in attitudes about suicide with males tending to possess more maladaptive attitudes about suicide than females [6]. These patterns are evident across development from adolescents to elderly adults [1,7], yet are generally more robust among individuals 15 to 29 years of age [2].

Taking into consideration such robust gender differences, one might expect that validated approaches to suicide prevention would also commonly consider gender differences when planning and evaluating the effects of interventions. Unfortunately, there has been insufficient attention to individual differences in risk for suicidality and intervention response [8]. Prominent reviews of suicide prevention literature have rarely focused on the role of gender [5,9]. Recently, this oversight has been identified by Klimes-Dougan, Klingbeil, and Meller [10], who called for further inquiry into the issue of gender differences in suicide prevention programming.

This paper serves as an illustrative literature review of existing empirical investigations that address the important question: Do males and females differ in their responsiveness to suicide prevention programming? Much of the available research did not consider gender differences [11,12,13,14,15,16]. Additionally, a few studies evaluated gender but failed to show gender differences [17,18,19,20]. In this review we highlight studies that find either a more favorable or less favorable response to suicide prevention efforts for males or females.

Adolescent suicide is a growing health concern calling for concerted investigation. This study emphasizes the development of gender-attuned adolescent prevention programming but also considers prevention strategies from a variety of age groups and settings. The hope is that by considering a wide array of approaches to suicide prevention, more general themes will emerge; themes that pertain to adolescent development and can be thoughtfully considered when (a) attempting to understand the mechanisms of suicide risk in youth; (b) modifying existing youth suicide prevention programs; and (c) planning new ways to intervene.

## 2. Method

**Figure 1:**
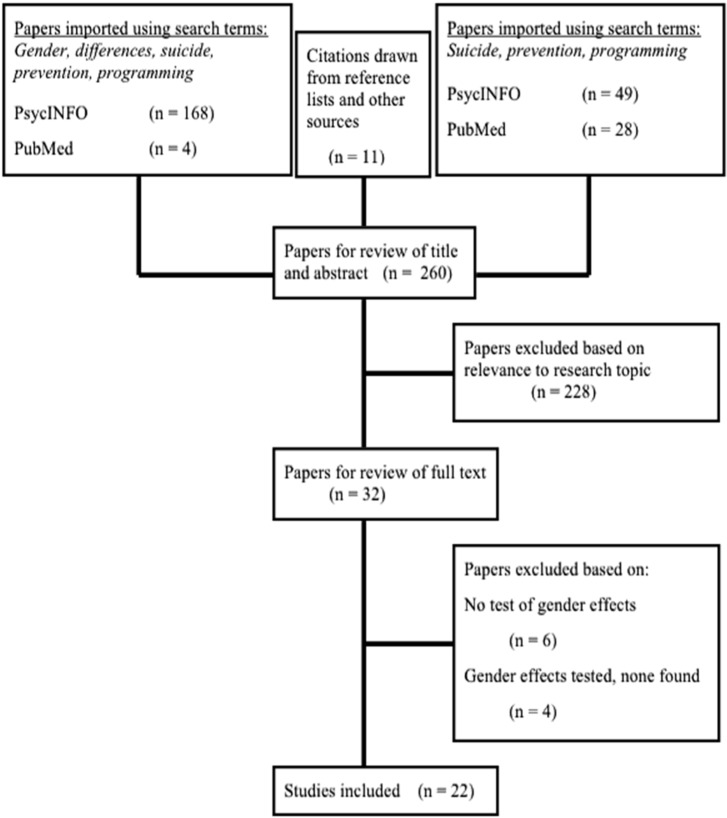
Flowchart of literature review process.

This review considered a broad array of published and peer-reviewed research that evidenced gender differences in the effectiveness of suicide prevention efforts. Figure 1 presents the process in which research was retrieved and either excluded or included for review. Studies that assessed utility of prevention strategies were only included if outcomes for males and females were specifically assessed and gender was found to moderate program success [21,22,23,24,25,26,27,28,29,30,31,32,33,34,35,36,37,38,39,40,41,42]. Research was retrieved through searching online databases (PsycINFO and PubMed) and utilizing the reference lists of relevant articles. The search terms used were *gender, differences, suicide, prevention,* and *programming*. Since the relevant literature retrieval resulting from using search terms was exhausted quickly, the terms *gender* and *differences* were omitted and the subsequent retrieved literature was individually read and scrutinized for mention of gender differences within the results and discussion sections. Two hundred sixty articles were preliminarily screened by title and abstract; of these, two hundred twenty-eight were excluded based on irrelevance to our research topic. The remaining thirty-two articles were read in full. Of these, ten were excluded because (a) gender differences were not tested [11,12,13,14,15,16]; or (b) gender differences were tested but none were found [17,18,19,20].

The remaining twenty-two studies evaluate common universal prevention programs as well as targeted programs. Because of the relative dearth of literature addressing gender differences in suicide prevention programming among the adolescent population within multiple settings, research selected for this review was non-discriminatory in that it considered a wide developmental window, although implications for adolescents are emphasized in the discussion section. The presentation of the results follows the categorization model proposed by Gould, Greenberg, Velting, and Shaffer [43], that popular prevention programming exists within and may be divided for analytic purposes into multiple settings: schools, communities, and healthcare systems.

## 3. Results and Discussion

### 3.1. School-Based Prevention Programming

School-based suicide prevention efforts are widely studied. The bulk of the existing research is conducted in high schools. The most common school-based prevention approaches include suicide awareness curricula, adult and peer gatekeeper training, and screening.

#### 3.1.1. Curricula

Typical goals of suicide prevention curriculum are to increase awareness of suicide risk and direct students to resources. Information sessions typically range from one to a few class periods. Action steps are provided for those encountering a friend struggling with suicide and supportive resources are distributed [44]. Much of the existing research uses a pretest-posttest design to evaluate knowledge gained after implementation of suicide awareness classes. The past several decades of research on curriculum development and evaluation have been critical in identifying important differences in male and female adolescents. Several studies have shown females to be more responsive to and accepting of suicide awareness programming in schools when compared to male classmates [24,25]. Females are also more likely than males to learn and employ adaptive behaviors following programming, such as seeking help for a suicidal friend [28,29,30].

Perhaps the strongest evidence of gender differences in response to curricula was reported in the work of Shaffer *et al.* [22]. A pretest-posttest design was used to evaluate a suicide prevention program in four high schools. The program consisted of a mixture of teacher instruction and discussion. Few positive effects of the program were found. Having participated in the intervention, male suicide attempters were significantly more likely to feel that the program “will make it harder to deal with my friends’ problems,” and were significantly more likely to “know someone who was upset a lot by the program.” More males than females who had attempted suicide and more male non-attempters found the programs boring. Attempters exposed to the program were less likely to recommend the presentation of the program to other students and were more likely to agree that talking about suicide “makes some kids more likely to try to kill themselves.” These results were highly controversial, leading some to question the utility of curriculum programs.

The only school-based curriculum to date that has documented decreased suicide attempts in program participants has also noted gender differences. Aseltine and DeMartino [29] assessed the Signs of Suicide (SOS) program, a two-part prevention program targeted at high school students. The objective of the SOS program is to reduce suicidal behavior by first educating participants on recognizing depressive symptoms in themselves and others using a curriculum, and subsequently administering a suicide screen. After a video and discussion session, a total of 1027 participants were evaluated on (a) self-reported suicide attempts and ideation; (b) knowledge and attitudes about depression and suicide; and (c) help-seeking behaviors. Females showed greater knowledge of and more constructive attitudes about depression and suicide than males. Females were also more likely than males to seek help for emotional disturbances, to intervene on behalf of peers, and to report their own suicidal thoughts or attempts. A replication of the original SOS study also found that males appeared to be less likely than their female counterparts to benefit from the SOS program [30]. That is, after exposure to the SOS program, females had greater knowledge, more adaptive attitudes, and higher rates of help-seeking when compared to males.

#### 3.1.2. Gatekeepers

Implementation of adult gatekeepers and peer helpers in schools are analogous strategies used to identify those at risk for suicide. Gatekeeper training may emphasize a surveillance model (*i.e.*, an increase in knowledge about suicidality allowing gatekeepers to effectively respond and make appropriate referrals) or a communication model (*i.e.*, fostering communication between students and gatekeeper to improve help-seeking behaviors) [45].

Eckert *et al.* [24] evaluated college students’ perceptions of a gatekeeper-training program. Participants rated (a) the program acceptability; (b) intrusiveness; and (c) the required time demands. A case scenario was described in which all school staff members receive training so that they could more aptly recognize suicidal behavior and refer at-risk adolescents to a school psychologist. Females rated gatekeeper programming as a significantly more acceptable means of suicide prevention when compared to their male counterparts [24]. There were no differences between male and female perceived intrusiveness and time demands.

Conversely, Ciffone [31] evaluated a gatekeeper-training program implemented in a suburban high school using a pretest-posttest design. The program took place in a health class and involved a presentation by a social worker, written handouts, a video filmstrip including a case presentation and examples of peer responses to suicide, a class discussion, and a self-esteem checklist. A survey sought to determine how likely the students were to take suicide threats seriously, seek help for suicidal thoughts for both themselves and their peers, and whether or not they would try to counsel a suicidal friend without telling an authority figure. There was a significant, desirable effect of the program on males that was not present for females to the question, “If I felt very upset, I would seek a mental health professional.” The author suggested male identification with the male character on the filmstrip (who died from suicide unlike the female, creating a sense of male urgency to seek help) and potential gender-biased questionnaire wording contributed to this unique finding [31].

#### 3.1.3. Screening

Suicide screening has received significant attention as a primary means of intervention. Typically, questionnaires are distributed school-wide and require that a student rate statements assessing recent and past suicide ideation. Screening is often multiphasic; besides completing self-report questionnaires, those endorsing suicidality may be interviewed by a mental health professional and could potentially be referred for treatment. In part, this approach was promoted as an alternative to the school curriculum [46].

Although in recent years the enthusiasm of suicide screening has been met with considerable resistance [47,48], Shaffer *et al.* [22] were initially strong advocates for screening. His group at Columbia University evaluated the impact of the Columbia Suicide Screen (CSS), in which 1729 high school students completed the CSS and Beck Depression Inventory (BDI) between the years of 1991 and 1994. Students endorsing risk items on the CSS were then administered the Diagnostic Interview Schedule for Children version 2.3 (DISC). A higher proportion of females screened positively for depressive symptoms and completed the DISC interview. Females who completed the CSS more often endorsed anxiety, irritability, unhappiness, and suicidality. Males more frequently endorsed substance use. It is unclear to what extent these reports accurately reflected gender differences. It is possible that while the Columbia Suicide Screen was efficacious in recognizing suicidal intent in females, males were not as transparent in reporting potential suicidal intent. Furthermore, there is a suggestion that males were less willing to complete the DISC interview after screening, which may translate to a perceived intrusiveness of the measure [22].

Within a college student sample, Garlow *et al.* [32] assessed the results of the American Foundation for Suicide Prevention College Screening Project at Emory University. A nine-item depression module measured suicidal ideation, past suicidal attempts, episodes of deliberate self-harm, and symptoms of distress. Although more females (*n* = 519) than males (*n* = 205) volunteered to participate in this study, a larger proportion of male respondents (14.6%) were willing to report suicidal ideation than females (9.83%). The authors hypothesized that the emphasis of depression in the screen may have caused the female-rich sample and suggested that an emphasis on anger or stress may have been more successful in attracting male participants [32].

### 3.2. Community-Based Prevention Programming

Community-based programs intend to direct any community member to available prevention resources. These programs follow the general pattern of either (a) implementing a primary prevention approach to the public; or (b) a secondary recognition of suicidal intent paired with referral and counseling. Mass-media campaigns and suicide prevention centers/hotlines are of the most widely publicized and researched community outreach programs to date [5]. While potentially applicable to youth, to date this line of research focuses primarily on adults.

#### 3.2.1. PSA and Media Campaigns

Public service announcements (PSAs) are developed to educate the public on issues of central relevance to suicide prevention. Although this approach has considerable large-scale appeal, it has also sparked controversy with some raising concerns about iatrogenic effects that the messages may provoke [33,49]. Most evaluations have taken place within the context of a community with billboard messaging or radio and television advertisements.

Daigle *et al.* [34] measured the efficacy of a multimedia-based prevention approach (via radio, television, and billboards) intended at reducing suicide rates among males in Quebec, Canada. They exclusively evaluated the results of this campaign on males from Suicide Prevention Weeks (SPW) in 1999, 2000, and 2001, by targeting a male audience (e.g., *Pain is not gender-specific—yet 80% of suicides are committed by men*). Male participants exposed to the campaigns gained more knowledge of suicide facts and resources, yet there was no evidence of an influence on males’ intention to seek help.

In the only study on PSAs with high school students, Klimes-Dougan *et al.* [33] examined the perceptions of television and billboard service announcements in a simulation study (44% male). Participants completed a post-test after having been exposed either to a suicide prevention billboard, a television advertisement, or no information. Overall, females were more knowledgeable about depressive symptoms and estimated higher rates of suicide ideation, attempts, and death. Males in the billboard condition were more likely than females to rate the billboard as not useful. Males in the billboard condition found other types of public service announcements to be more useful, such as television advertisements and pamphlets. The authors concluded that healthy females (defined by those void of depressive symptoms) were found to be most likely to benefit from suicide prevention public service campaigns. A similar simulation study conducted with a group of primarily female college students (*N* = 862) showed a main effect for gender [35]. Across all conditions, females were more knowledgeable about depression, endorsed more adaptive help-seeking attitudes, and endorsed fewer maladaptive attitudes.

#### 3.2.2. Crisis Centers/Hotlines

Crisis centers and hotlines have been met with mixed interpretations regarding actual success in suicide prevention [50]. The guiding principle of suicide prevention centers holds that individuals are more likely to consider suicide when they have had a major traumatic event or crisis. These resources are thus available to address these more acute needs.

A study by Medoff [36] utilized 1979 United States census data to extract information regarding the effectiveness of suicide prevention centers in reduction of mortality rates. Males constituted a much larger proportion of lives saved in the presence of suicide prevention centers for that year (2821 as compared to 789, respectively). The results indicated that each additional implemented suicide prevention center reduced the state’s suicide rate for white males aged 15–64 by 3.7 suicides per 100,000 people, while for white females, 1 per 100,000 people. These findings may be directly based on the aforementioned fact that males tend to have higher rates of lethal suicide attempts [1,7], but nevertheless suggests a significant benefit of suicide prevention centers for white males.

A study by Miller, Coombs, Leeper, and Barton [37] compared several central city counties in the United States in a) suicide mortality rates, and b) presence and frequency of suicide prevention centers, crisis centers, and mental health facilities. Using data from the National Center for Health Statistics, the authors focused on regions that had recently initiated prevention centers *versus* counties in which the number of centers remained the same. They found that the implementation of suicide prevention centers favorably impacted and minimized suicide rates in white females younger than twenty-four years of age, the most frequent callers to crisis centers. Counties in which the number of centers remained stagnant housed increased mortality rates for both males and females [37].

### 3.3. Health-Based Prevention Programming

Suicide prevention strategies in healthcare systems primarily operate by general screening of all patients during clinic visits. A form of gatekeeper training may be dually employed to train general practitioners and specialists to recognize and act on potential patient suicidality. Screening at clinics is a promising prevention strategy since most individuals who die by suicide have had contact with a primary care physician within one month of death [51]. Several studies have shown that females benefit from health-based suicide prevention over males [38,39,40]. This is not likely to be due to the nature of the intervention per say, but rather due to the increased likelihood of females having contact with their physician and reporting suicidal intent to or being recognized as suicidal by their physician.

The frequently cited study by Rutz, Von Knorring, and Walinder [41] found that after implementation of an intensive training program intended to improve general practitioner’s recognition of depression and suicidality in Gotland, Sweden, adult suicide rates vastly declined. The decrease in suicide was due to an almost complete reduction in female suicides; the rate of male suicides remained stagnant [41]. Several hypotheses for the disparity were presented in follow-up research: that depressed men present with externalizing behaviors rather than internalizing and that depressed men are inherently “incapable for asking for help or showing weakness” [52]. Although research conducted after the project ended found that suicide rates had returned to baseline levels [53], the original authors have bolstered their findings by coining the “male depressive syndrome” and creating the Gotland Male Depression Scale [54].

Follow-up care may also be important. Postcards from the EDge, a project instituted in New South Wales, Australia, used a simple design of sending postcards to patients who had attempted suicide by deliberate self-poisoning with the hopes of preventing repeat episodes. Carter *et al.* [21] conducted a randomized controlled trial in which eight postcards were sent throughout the year to patients 16 years of age and older. The postcards were addressed from the hospital and included phrases such as, “we hope things are going well for you”, and “if you wish to drop us a note we would be happy to hear from you” [21]. The intervention was effective in reducing the risk of repeat, deliberate, self-poisoning in females, but not in males. A follow-up with the same sample at 24 months post-episode found similar results; the intervention was effective for females but not for males [42].

### 3.4. Discussion

There is currently a wide range of suicide prevention strategies employed in the school, the community, and within primary care settings. A pattern of enhanced benefit for females is evident across approaches, from school curricula [25,27], to PSAs [33], to screening efforts employed at primary care settings [41].

First, within school-based programming, females seem to be more willing to engage in communication, gain more knowledge from curricula, and show more concern for at-risk peers [26,29]. Males seem to be more reluctant to disclose self-injurious behaviors and do not favor active involvement in suicide awareness curricula [23,25,28]; however, one study provided evidence that males can benefit from school-based programming [31]. Anonymity in divulgence of suicidal intent may present a more appealing means of outreach. School-wide screening may be promising in recognizing self-injurious behavior in men [32].

Next, community-based prevention programming produce mixed results. Males can be receptive to certain types of PSAs [34], yet females’ increased willingness to utilize prevention centers and higher likelihood to benefit from PSAs translate to more positive outcomes in preventing suicidal behavior [33,36,37]. There appear to be conditions required for community-based approaches to produce beneficial outcomes in suicidal individuals of both sexes; for example, race/ethnicity [36,37] and type of content in PSAs [33].

Finally, although efforts have been made to evaluate healthcare-based suicide prevention, in very few cases were gender differences directly reported, yet most showed females maximally benefitting [21,41,42]. It should be noted, however, that willingness to seek help contributes in large part to female success: higher representation of females seeking care in health-based settings precedes diagnosis, treatment, and recovery [38,39,40,55].

#### 3.4.1. Implications for Suicide Prevention in Adolescents

There are some broader societal patterns that may be relevant to gender differences in adolescent suicide prevention. Help-seeking attitudes and behaviors show some of the most robust differences for young males and females [56,57]. It is likely that early rearing plays a role in the perceptions of suicide prevention strategies and help-seeking, with such patterns perpetuating into adolescence and early adulthood [56,58]. For example, the masculine culture of self-reliance and stoicism may help explain why males repeatedly rate programming requiring self-disclosure as “ineffective” or “not useful” [24,28]. Conversely, the female culture of tight-knit collectivism and cathartic emotionality enables females to cope with stress and anxiety by venting and communicating with family, peers, and other adults [56].

These broader socialization patterns may be reflected more specifically in youth suicide prevention outcomes. Indeed, available research consistently shows that adolescent females are more likely than males to seek both informal and formal support for emotional disturbances [56,57,59]. Males hold maladaptive attitudes toward suicide prevention services and seeking professional help [28,43]. Additionally, much of Emile Durkheim’s work brings to light the social underpinnings of suicide [60]. Certainly there are socioenvironmental conditions beyond masculinity *versus* femininity at play that are driving a small proportion of depressed suicide attempters to completing suicide [1]. The interplay between (a) the vast gap separating suicide attempters and completers; and (b) the gap between male and female suicide completions, calls for further research, particularly into potential factors mediating gender and suicide completion.

Individuation of interventions poses a promising strategy to counter overindulgence in using gender roles to tailor suicide prevention programming. Perhaps males may be most able to benefit from programs in which choice in type, length, or nature of involvement is maximized, while the perceived help-seeking obligation is minimized. Now that we know gender can moderate intervention response, the question of how this information can be used to reach the adolescent population remains. Of course, adaptation of this sort would be more or less amenable to certain programs. For example, health classes in schools can be adapted to consider different approaches for males and females. With PSAs it is more challenging to control who is being reached. Nevertheless, PSAs have traditionally been directed toward a specific audience [34], and methodically placing PSAs in gender and adolescent appropriate environments (*i.e*., male restrooms, men’s magazines, gaming or entertainment websites such as The Chive) has been suggested [61]. The risk of iatrogenic effects in gender-tailored public service announcements must be considered, however, as broader efforts aimed at increasing public knowledge of suicide has resulted in suicide influx [33,49]. In terms of health-based settings, a recent study by Hernandez, Oliffe, Joyce, Söchting, and Ogrodniczuk [62] found through self-report measures that when given the option, outpatient men, similar to outpatient women, preferred individual psychotherapy to other treatments (i.e. medication and no treatment/wait and see).

It seems plausible that interventions preferred by males would be more effective in meeting their needs. Preference-based approaches can highlight individual differences, backgrounds, and experiences, and offer a solution to minimize iatrogenic effects. Allowing the participant to select a preferred type of programming can also help them to feel invested in the programming. The idea of preference-based prevention is consistent with the empowerment perspective [63], which suggests that fully engaged participants are active decision-makers in their intervention. Furthermore, personal control over treatment has led to better patient outcomes in several studies on depression [64,65,66]. Although there is a push to encourage treatment and programming facilitators to provide options, preference-based interventions have yet to be methodically implemented in suicide prevention efforts. We currently know little about the preferences of adolescent males; future research would benefit from piloting preference-based approaches in schools and adolescent treatment facilities. By ensuring personalization in suicide prevention programming, mental health professionals can anticipate greater receptiveness and, conceivably, a decrease in nationwide youth suicide attempts and completions.

## 4. Conclusions

The results of this review point to the priority for tailoring prevention programs so that adolescents, a high-risk group for suicidal behavior, can maximally benefit. Currently, females appear to be the primary beneficiaries of several prevention efforts, while males more often exhibit deleterious effects from exposure to programming. Concerted effort is needed to promote strategies that will also benefit males. A fruitful direction may be to separately implement and administer programming to males and females. For hard-to-reach males, preference-based programming allows an element of independence. Nevertheless, as we consider such robust gender differences in receptiveness to suicide prevention programs, the need for individualized strategies is drastically apparent.
